# The Smoke Pall

**Published:** 1920-05-29

**Authors:** 


### THE SMOKE PALL.

### Paradise for Painters and Laundries.

The cost of the poison and smoke in the atmosphere of the large towns and cities cannot be so strictly expressed in pounds, shillings, and pence in relation to health as in relation to the laundry bill, the up-keep of property, and the time spent over the washtubs. Leeds has 17 per cent. less sunshine than country districts four miles beyond its boundaries. Doubtless similar comparisons could be gathered from Sheffield and elsewhere. Manchester spends 7½d. per house a week more than Harrogate on washing materials, and stands over the washtub an extra amount of 668 years every year, merely because of the greater smoke density over the city. It is not to be wondered at

**THE PUBLIC WELL-BEING**—(*continued*).

that the Manchester Corporation displays some energy and enterprise in fighting these disagreeable and unhealthy circumstances. Under the Health Committee there is an Air Pollution Advisory Board, which has presented a remarkable report giving in statistical form the cost of damage done by smoke. The Committee followed the lines adopted in Pittsburg, U.S.A., where it was found the damage was equivalent to £4 per head of the population per annum. Questionnaires were issued by the Manchester Committee to all sorts and conditions of men—superintendents of hospitals, householders, architects, house painters, property owners, etc. The house painters revelled in the smoke so far as they were concerned, and a typical answer from them was, "As a painter he would be pleased if the smoke did even more damage." Indeed, the answers made it clear that Manchester is a painter's paradise. A number of architects estimated that a clean atmosphere would reduce the cost of the upkeep and maintenance of buildings by as much as 20 per cent. Others, more cautious, said from 15 per cent. to 20 per cent., but none denied that considerable saving would be effected. Property owners were asked to estimate the yearly cost of outside painting and repairs in the case of a residential property in the city rented at £75 per annum, and to compare it with the cost which would be incurred in the case of a similar residence in the country. Several stated that the cost in the city would be 50 per cent. greater; others 25 per cent., and one 10 per cent.

Twenty-five hotel proprietors in Manchester replied to the questions. One estimated that 100 per cent. would be saved on inside and outside decorating and painting if the hotel were situated in a cleaner atmosphere. From one of the replies it appears that "curtains last only half as long as in Blackpool"; from another that they last "only three-quarters as long as in London, Liverpool, and Birmingham."

### Homely Illustrations.

"White silk curtains washed once a fortnight in Manchester, in Aberdeen once a year."

"Compelled to wear dark colours, whereas light ones are preferred."

"When making purchase, question is—will it show the dirt soon?"

"Personal linen in Strines last clean three days as compared with one in Manchester. One hour's walking in the streets of Manchester will soil a collar as much as a whole day at Buxton." "Am of opinion that Manchester is the dirtiest city in England. Collars become dirty in a day, whereas on the Continent they will last a week."

"Few building materials will stand the smoke and acidity of the Manchester atmosphere."

"Many men will not visit Manchester except under dire necessity on account of bronchial trouble."

The replies altogether, while they threw a good deal of direct light on the extent of the smoke evil, yet fell short of statistical exactitude. The Committee, aiming at material on which it could base an *actual* estimate, then undertook a comparative inquiry, with the object of obtaining an estimate of what may be called washtub losses, or what Manchester pays for household and personal laundry over and above what is paid by a community with the same standards of life, but living in a purer atmosphere.

The services of one of the public health inspectors of the Corporation were placed at the disposal of the Committee. Her instructions were to obtain one hundred exact and comparable statements for Manchester and Harrogate respectively as to the cost of the weekly washing in working-class houses. As far as possible she visited houses of the same class in both towns; the average weekly rent of the houses visited in Manchester was 5s. 11d., and in Harrogate 5s. 9d.

The investigator's returns, the report states, show that, on an average, the weekly wash was one hour longer in the performance in Manchester than in Harrogate. There are in Manchester 112,616 small houses where the housewives, as a general rule, do their own housework single-handed. If they all take one hour longer each week to do the washing than they would do if they lived in a clean town, the sum total of time wasted in the course of a year is equivalent to 5,850,000 hours, or 668 years. This is the penal sentence which the housewives of Manchester, who do their washing at home, serve each year for black smoke!

The inquiry showed that, as between Manchester and Harrogate, the extra cost of the Manchester wash in materials and fuel was 7½d. a week per household. An extra cost of 7½d. per week is equal to a loss of £1 12s. 6d. per household per annum, and for the 112,616 families living in houses at 11s. a week and under—*i.e.*, those where housewives do their own washing—this amounts to a loss of £183,000 per annum.

### The Black Smoke Tax.

For the purpose of arriving at an estimate of the loss on household washing for the whole city it has been assumed that the loss in the larger households is the same as in the smaller ones, which is an extremely conservative consumption. On this basis the loss for the larger houses amounts to £59,705, making, with £183,000 for the families living in smaller houses, a total loss of £242,705 per annum. These figures take no account of the added wear and tear entailed by the extra washing involved.

Even on so cautious an estimate as the foregoing, it is pointed out, the direct taxation levied by black smoke in Manchester amounts to practically a quarter of a million sterling. The cost of household washing is only one item amongst many; the damage to health, buildings, merchandise, decorations, and vegetation would greatly swell the total. It is quite clear, therefore, that the sum of three-quarters of a million pounds (equalling £1 per head of the population), which was the figure obtained by the Hon. Rollo Russell while investigating the conditions obtaining in London, and which has up to the

THE PUBLIC WELL-BEING—(*continued*).

present been considered a reasonable estimate of the annual cost of the damage done by smoke in the various large cities of England, was well within the mark, specially when the estimate of £4 per head of the population of Pittsburg is borne in mind.

In conclusion, it is urged that it would well repay Manchester to expend a large amount of thought and money on any measures that would help to reduce its enormous yearly smoke tax.

There is gathering a solid move against the domestic grate as a first-class offender. It is to be supposed, however, that we shall have to wait a long while before that comforting and cosy nuisance is eradicated. But the health bill against it must be enormous.

